# Long-Term Outcomes of External Repair as a Rescue Operation for Atrioventricular Groove Disruption following Mitral Valve Surgery

**DOI:** 10.1186/1749-8090-10-S1-A150

**Published:** 2015-12-16

**Authors:** Nikola Dobrilovic, Jaishankar Raman, James G Fingleton, Andrew Maslow, Arun K Singh

**Affiliations:** 1 Division of Cardiovascular and Thoracic Surgery, Brown University, Providence, RI, 09205, USA; 2 Department of Cardiovascular and Thoracic Surgery, Rush University, Chicago, IL, 60612, USA

## Background/Introduction

Atrioventricular (AV) groove disruption is a lethal complication of mitral surgery. Traditional teaching mandates that an "internal" repair be performed requiring prosthesis explantation, complete atrioventricular groove reconstruction utilizing a patch positioned from within the cardiac cavity, and subsequent prosthesis re-implantation. This is a massive undertaking and yields uniformly poor results.

## Aims/Objectives

We examine the utility of an alternative, "external" approach for rescue of atrioventricular groove disruption.

## Method

The study was conducted as a retrospective review of a multi-surgeon, multi-institutional experience inclusive of all patients suffering disruption of the atrioventricular groove following mitral surgery. All patients experiencing this complication (consecutively and exclusively) underwent "external" repair as a rescue procedure. The external repair technique was conducted on cardiopulmonary bypass support using direct (felt pledget/strip reinforced) suturing of the AV groove. This was supplemented as needed with applications of BioGlue, external bovine patch, left atrial patch suture technique, and coronary bypass of the circumflex system.

## Results

Over a span of 20 years, 3071 mitral valve surgeries resulted in 13 AV groove disruptions (incidence 0.42%). Average patient age was 75.2 years (range 59-90), 77% (10/13) were female. Thirty-day mortality was 15.4% (2/13). Hospital mortality was 23.1% (3/13). Hospital stay (day of surgery to day of discharge) averaged 27.6 (range 8-42) days. Patient follow-up averaged 5 years (range 3 months to 10 years). One-, three-, and five-year survival rates were 72.7%, 72.7%, and 44.4%. Seven patients are currently still alive and remain in (no greater than) NYHA Heart Failure Class II, with an average follow-up of 4 years (range 3 months to 8 years). One patient developed a large chronic pseudoaneurysm originating from the AV groove repair site identified after discharge as an outpatient.

## Discussion/Conclusion

The described "external" approach represents an effective, alternative repair technique for rescue of otherwise lethal atrioventricular groove disruption.

